# A practice-based analysis of combinations of diseases in patients aged 65 or older in primary care

**DOI:** 10.1186/1471-2296-15-159

**Published:** 2014-09-23

**Authors:** Pauline Boeckxstaens, Wim Peersman, Gwendolyn Goubin, Souhila Ghali, Jan De Maeseneer, Guy Brusselle, An De Sutter

**Affiliations:** Department of Family Medicine and Primary Healthcare, Ghent University, Ghent, Belgium; Community Health Centre Botermarkt, Ghent, Belgium; Community Health Centre De Sleep, Ghent, Belgium; Department of Internal Medicine, Ghent University Hospital, Ghent, Belgium

## Abstract

**Background:**

Most evidence on chronic diseases has been collected for single diseases whereas in reality, patients often suffer from more than one condition. There is a growing need for evidence-based answers to multimorbidity, especially in primary care settings where family doctors (FD’s) provide comprehensive care for a high variety of chronic conditions. This study aimed to define which disease and problem combinations would be most relevant and useful for the development of guidelines to manage multimorbidity in primary care.

**Methods:**

A practice-based cross sectional analysis of clinicians’ chart reviews in 543 patients aged over 65 registered within two family practices in Ghent, Belgium. Main outcome measures were prevalence of disease and problem combinations and association strengths.

**Results:**

The prevalence of multimorbidity (Cumulative Illness Rating Scale >1) in the study sample is 82.6%. The most prevalent combination is hypertension-osteoarthritis (132/543). Moderate to strong associations (Yules Q > 0.50) are reported for 14 combinations but the corresponding prevalences are mostly below 5%. More than half of these associations show a contribution of a psychiatric problem or a social problem.

**Conclusions:**

This study confirms the high prevalence of multimorbidity in patients aged over 65 in primary care. Hypertension-osteoarthritis is defined as a frequent combination however 94% of these patients have more than two disorders. The low prevalence of specific combinations, the high prevalence of psychiatric and social problems and the general complexity of multimorbidity will hamper the usefulness of randomized trials or guidelines at practice level. There is a need to explore new paradigms for addressing multimorbidity.

## Background

Multimorbidity is clearly on the rise [1, 2] and a challenge for clinical practice [3, 4]. In family practice, patients with multimorbidity are the rule rather than the exception [5]. Yet, most available evidence to treat chronic diseases has been collected in single disease trials, often excluding patients with comorbid diseases. Clinicians feel a growing need for evidence that can be applied to patients with multiple diseases [6, 7]. A possible solution to tackle this evidence gap may be to look for specific disease combinations with a high prevalence and to engage in the development of randomized clinical trials or guidelines on patients with these combinations of diseases [8, 9]. Few studies have focused on how diseases co-occur. Mostof these studies assess comorbidity instead of multimorbidity. Comorbidity implies an index disease (e.g. Chronic Obstructive Pulmonary Disease (COPD) and comorbidity) whereas multimorbidity is defined as any co-occurrence of medical conditions within a person. From the perspective of primary health care multimorbidity is more relevant because general practitioners deal with the broad spectrum of the morbidity of the patient without prioritizing specific disease categories. In order to fill the evidence-gap for multimorbidity we need to focus on those problems that influence clinical management at the patient level [3, 4]. However, most studies use large databases from population surveys or automated extraction of disease codes out of medical records or insurance claims. Datasets based on automatized extraction of disease codes do not necessarily identify the problems that are relevant at the point of care [3]. They might also lead to over- or underestimation of diseases. For example, a disease code on low back pain might relate to a patient that only consulted for advice but the same disease code could also relate to a patient with severe impairment, decreased quality of life or intensive need for physiotherapy. This study aims to identify the problems that influence clinical management at the patient level in primary care in order to identify combinations of problems that could be a relevant focus for trials or guidelines for patients with multimorbidity.

## Methods

### Subjects

We conducted a practice-based cross sectional analysis of the patient records of all patients aged 65 or older who were registered in two community health centers (CHC) in Ghent, Belgium. CHC’s provide interdisciplinary comprehensive primary health care using a capitation payment system accessible for all people residing in the area covered by the CHC. The choice for CHC’s was based on the fact that the capitation based system enhances continuity of care and consequently leads to more complete information in patients’ medical records. The CHCs with the largest patient lists were chosen for inclusion of patients.

### Chart review

Medical records in the participating practices are based on the problem-oriented medical record model as proposed by Weed (1968) [10] in which the patient's history, physical findings, laboratory results, etc. are organized around patient’s problems. These medical records include a list of all the problems of the patient including both clearly established diagnoses (such as diabetes II or COPD) and other problems relevant for patient management in primary care (such as “symptom diagnoses”, social problems,…). The underlying classification used in these records is the International Classification of Primary Care (ICPC) [11] For each problem presented by the patient, information is registered in the SOAP-format (subjective (S), objective (O) assessment (A) diagnostic and treatment plans (P). Three family doctors (PB, GG and SG), who were part of the medical staff of the participating practices, performed a detailed clinical review of each electronic patient record (EPR) and its additional paper file. They assessed whether or not a problem was of influence on the management for that patient. For each patient this resulted in a list of all problems with clinically relevant impact, including social problems and relevant medical history. To provide an estimation of the prevalence of multimorbidity in the study sample all patients were allocated a Cumulative Illness Rating Scale (CIRS) score based on the scoring guidelines published by Hudon [12–14]. To harmonize the clinical assessment, data extraction and allocation of the multimorbidity scores, 30 patient records were independently reviewed by the three family doctors who performed the assessment and results were compared and discussed in a meeting to attune the assessment approach. Every patient’s problem list was anonymized and transferred into a separate database for analysis. In order to find a reasonable way for including all relevant but often low prevalent problems into a sensible analysis of combinations a process of summarization was performed in which the research team constructed a list of 23 problems (Table 1). In this list different levels of detail were used: some disorders like COPD, diabetes and depression were analyzed at disease level, disorders like osteoarthritis and cardiac rhythm disorders were considered as “diagnostic group” and other disorders were summarized at the level of a body system (eg the EENT (Eye Ear Nose and Troat) system and the neurological system). Although they are not considered in the CIRS social problems were also included because of their possible relevance to the patients’ clinical management at the point of care.

**Table 1:** **Prevalence of the 23 problems selected for further analysis of combinations**

Problem	Prevalence%	n
Hypertension	48,4%	263
Osteoarthritis	47,3%	257
EENT (eye, ear, nose, throat) system	28.5%	155
Psychiatric system	20.6%	112
Neurological system	19.8%	108
Upper gastrointestinal system	14.5%	79
COPD (Chronic Obstructive Pulmonary Disease)	14,2%	77
Diabetes	14,2%	77
Ischemic heart disease	14,2%	77
Lipid disorder	14,0%	76
Social problem	14.0%	76
Cardiac rhythm disorder	13,3%	72
Overweight	12%	65
Heart failure	11%	60
Renal system	9.3%	52
Nicotine abuse	9,4%	51
Lower gastrointestinal system	8.3%	45
Depression	7,7%	42
TIA (transient ischemic attack)/CVA (cerebrovascular accident)	7,2%	39
Cancer	7,2%	39
Osteoporosis	6,8%	37
Hepatic system	3.5%	19
Alcohol abuse	2,9%	16

### Analysis of combinations

To reveal the combinations which occur most frequently, prevalences were calculated for all possible combinations of these 23 problems. Next to the prevalence, Yules Q was used to measure the strength of the association for each possible combination. Yule’s Q is a symmetric measure taking on values between −1 and +1. One implies perfect negative or positive association, 0 (zero) no association. Yules Q measures > 0,70 represent strong associations and Yules Q measures < 0,70 and > 0,50 represent moderate associations [15]. A percentile bootstrap [12] procedure with 1000 data sets was used to estimate the 95% confidence interval for the Yules Q coefficients. All analyses were supported by SPSS version 19.0.

### Ethical approval

Ethical approval was provided by the Ethics Committee of Ghent University Hospital. The Belgian registration number of the approval for the data collection performed by GG and PB is B67020108605 and for the data collection of SG is B67020108596.

## Results

### Study population

**Figure 1:**
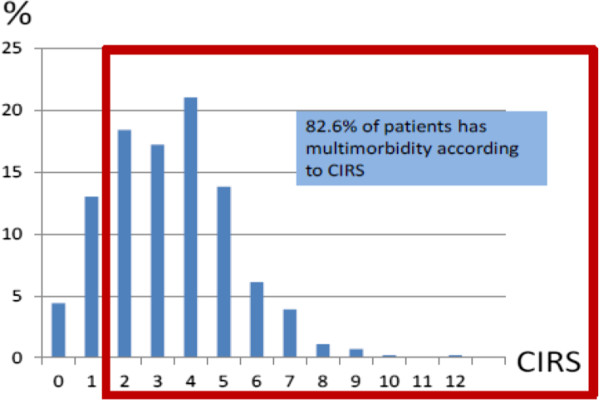
**Prevalence of multimorbidity according to the CIRS.**

On 1^st^ of October 2009, the practices under study listed 543 patients aged 65 or older. The mean age of patients in the study sample is 73 years (range 65–97), 47,3% of patients are male and 4,4% have no chronic disease (CIRS =0). The prevalence of multimorbidity (CIRS > 1) is 82.6% and 64.2% of the patients included have a CIRS score >2 (Figure 1). The most frequently affected CIRS domains are the endocrine (47,4%) and the musculoskeletal system (47%) followed by the cardiac system (30,4%). The most frequent disorders are hypertension (48,4%), osteoarthritis (48,4%), COPD (14,2%), ischemic heart disease (14,2%) and diabetes (14,2%). Table 1 reports the prevalence for the 23 problems which were used for further analysis of combinations.

### Combinations with a prevalence >5%

Table 2 shows all 39 problem pairs which occur frequently (prevalence > 5%). The most prevalent combination is hypertension-osteoarthritis (n = 132 Yules Q 0.11 CI : −0.06-0.28). More than 2 out of 3 of these combinations include hypertension or osteoarthritis and only 4 of these combinations are moderately or strongly associated (Yules Q > 0.50) : hypertension-renal system (Yules Q 0,52), osteoarthritis –lower gastro-intestinal system (Yules Q 0,54), psychiatric system – social problem (Yules Q 0,66) and diabetes –overweight (Yules Q 0,74).

**Table 2:** **Combinations with a prevalence over 5%**

Problem combination	Prevalence%	n	Yules Q (CI)
Hypertension - Osteoarthritis	24.3%	132	0.11 (−0.06-0.28)
Hypertension -EENT	17.0%	92	0.30 (0.12-0.46)
Hypertension - Psychiatric system	11.0%	60	0.13 (−0.08-0.32)
Hypertension - Neurological system	10.1%	55	0.06 (−0.15-0.27)
Hypertension – Diabetes	8.8%	48	0.32 (0.07-0.55)
Hypertension - Lipid disorder	8.7%	46	0.28 (0.04-0.49)
Hypertension - Upper gastrointestinal system	8.5%	46	0.28 (0.04-0.47)
Hypertension - COPD	7.9%	43	0.17 (−0.07-0.40)
Hypertension - Overweight	7.9%	43	0.39 (0.13-0.61)
Hypertension - Heart failure	7.7%	42	0.47 (0.21-0.67)
Hypertension – Social problem	7.7%	42	0.16 (−0.10-0.38)
Hypertension - Rhythm disorder	7.4%	40	0.16 (−0.10-0.40)
Hypertension - Renal system	7.0%	38	0.52 (0.26-0.74)
Hypertension - Ischemic Heart Disease	6.6%	36	−0.04 (−0.29-0.18)
Osteoarthritis - EENT system	15.8%	86	0.22 (0.04-0.40)
Osteoarthritis - Psychiatric system	12.1%	66	0.29 (0.08-0.48)
Osteoarthritis - Neurological system	10.3%	56	0.11 (− 0.10-0.32)
Osteoarthritis - Upper gastrointestinal system	9.2%	50	0.36 (0.15-0.57)
Osteoarthritis – Social problem	8.5%	46	0.30 (0.07-0.51)
Osteoarthritis - Lipid disorder	7.5%	41	0.15 (−0.08-0.37)
Osteoarthritis - Rhythm disorder	7.5%	41	0.22 (−0.03-0.46)
Osteoarthritis - overweight	7.4%	40	0.32 (0.07-0.55)
Osteoarthritis - ischemic heart disease	7.0%	38	0.05 (−0.21-0.29)
Osteoarthritis – COPD	6.4%	35	−0.04 (−0.26-0.19)
Osteoarthritis – diabetes	6.3%	34	−0.07 (−0.32-0.14)
Osteoarthritis - Lower gastrointestinal system	6.1%	33	0.54 (0.27-0.75)
Osteoarthritis - Heart failure	5.2%	28	−0.01 (−0.29-0.25)
Osteoarthritis - Depression	5.2%	28	0.41 (0.11-0.67)
EENT system - Neurological system	9.2%	50	0.46 (0.27-0.61)
EENT system - Psychiatric system	6.6%	36	0.11 (−0.15-0.33)
EENT – Social problem	5.7%	31	0.31 (0.07-0.52)
EENT system - Upper gastrointestinal system	5.5%	30	0.25 (−0.01-0.47)
EENT system - Rhythm disorder	5.3%	29	0.31 (0.05-0.53)
EENT system – COPD	5.1%	28	0.21 (−0.06-0.43)
Psychiatric system – Social problem	6.8%	37	0.66 (0.49-0.80)
Psychiatric system - Neurological system	5.7%	31	0.27 (0.03-0.47)
Psychiatric system - Upper gastrointestinal system	5.1%	28	0.43 (0.17-0.62)
Cataract- Hypertension	5.7%	31	0.35 (0.07-0.61)
Diabetes - Overweight	5.2%	28	0.74 (0.57-0.84)

CI = Confidence Interval. n = proportion and number of patients with this combination.

### Combinations with a strong or moderate association

Table 3 shows all 14 combinations with a strong or moderate association (Yules Q > 50). Diabetes-overweight (Yules Q : 0,74; CI : 0,57-0,84; n = 28) and nicotine abuse-alcohol abuse (Yules Q : 0,73; CI : 0,48-0,98; n = 6) emerged as the most strongly associated combinations (Yules Q >0,7). Social problems, psychiatric issues and locomotor problems (osteoarthritis and osteoporosis) are well represented within the list of combinations with a moderate association (Yules’ Q 0,5-0,7). Most of these associations (9/14) have prevalences below 5%.

**Table 3:** Moderate (Yules Q 0.50-0.70) and strong associations (Yules Q > 0.7)*

Problem combination	Yules Q	(CI)	Prevalence%	n
Diabetes –overweight	0.74	(0.57-0.84)	5.1%	28
Nicotine abuse – alcohol abuse	0.73	(0.48-0.98)	1.1%	6
Social problem – psychiatric system	0,66	(0.49-0. 80)	6.8%	37
Heart failure – renal system	0.60	(0.30-0.78)	2.8%	15
Depression – osteoporosis	0.59	(0.14-0.79)	1.5%	8
Social problem – osteoporosis	0.58	(0.25-0.77)	2.4%	13
Depression – upper gastrointestinal system	0.58	(0.28-0.76)	2.8%	15
COPD – nicotine abuse	0.56	(0.27-0.74)	3.1%	17
Osteoarthritis – lower gastrointestinal system	0.54	(0.29-0.74)	6.1%	33
Osteoarthritis – osteoporosis	0.53	(0.24-0.80)	5.0%	27
Social problem – depression	0.52	(0.27-0.74)	2.6%	14
Hypertension – renal system	0.52	(0.26-0.74)	7.0%	38
Osteoporosis – Upper gastrointestinal system	0.52	(0.12-0.73)	2.2%	12
Cancer – social problem	0.50	(0.14-0.71)	2.2%	12

*only significant results (p < 0,05 are reported).

## Discussion

This study aimed to include those disorders that influence the clinical management of patients and used clinician chart review to do so. By means of a practice-based analysis of individual patient records it was able to assess which combinations would be most relevant for the development of guidelines useful at practice level in primary care. Hypertension-osteoarthritis was identified as a most prevalent combination of diseases (24,3% of the study sample) but the association was not significant. The significant associations described in Table 3 generally show very low prevalence. In general this study indicates that the usefulness of RCT’s on disease combinations will be hampered by low prevalence at practice level, low association strengths and the fact that many patients present with more than 2 problems (64.2% of the study sample has a CIRS score > 2).

The combination hypertension-osteoarthritis has been described previously as the most prevalent combination in older persons [14]. Building evidence to manage patients with this combination could be useful because non-steroidal-anti-inflammatory-drugs (NSAIDs) might interfere with blood pressure control and keeping up an active life style can be difficult when suffering from osteoarthritis. Most other combinations with a considerable prevalence (which we have set at >5%) often include diagnostic groups (e.g. rhythm disorders) or body systems (e.g. neurological system) to which guidelines are not directly applicable. The combinations hypertension-diabetes, hypertension-lipid disorder, hypertension-overweight, hypertension-ischemic heart disease, hypertension-heart failure and diabetes-overweight are quite well covered within disease-specific guidelines because these combinations concern well established comorbid conditions based on causal associations [16, 17]. Many of the other combinations include osteoarthritis. Despite the existence of osteoarthritis guidelines [18], a standardized approach of the disorder is hampered due to the differing localization and the varying impact of the disorder on functional limitations and quality of life. Other combinations with considerable prevalence include mental health problems and social problems which is not only related to the high prevalence of both problems in this sample (psychiatric disorders (20,6%) and social problems (14,0%)) because remarkably, these problems are also highly represented in the associations with a moderate to strong correlation (Table 3). The importance of psychiatric comorbidity has been described previously [19] but to our knowledge there were no studies including social problems. From the perspective of clinical care psychosocial problems are very relevant as they can hamper compliance to disease specific guidelines for diabetes, heart failure,… [6,25] both in patients and providers. Qualitative inquiries in family doctors have also described them to be the main challenges within the management of multimorbidity [7].

### Multimorbidity is not limited to a specific set of chronic conditions

Our study confirms the low prevalence of specific combinations described in earlier studies and highlights the heterogeneity of multimorbidity described in other populations [19–22]. The study of Van Oostrom et al. [19] selected 9 chronic diseases for analysis of disease pairs with 29 other disease codes. Only one combination (depression-anxiety) was found to have a prevalence over 5% and 70% of patients had a comorbid disease which was not included within the nine most common chronic conditions. Despite the fact that Van Oostrom et al. included a younger population (patients aged over 55) and provided a less comprehensive assessment of multimorbidity, their main results are in line with our findings showing a wide variety in multimorbidity which is not limited to a specific list of chronic conditions.

### Tailored instead of standardized care

Our results have also clearly indicated that the usefulness of guidelines on disease combinations will not only be hampered by the low prevalence of the combinations, but even more by the fact that 94% of the patients with hypertension and osteoarthritis had additional problems and 64.2% of the patients in this sample had a CIRS > 2. Van den Bussche et al. [21] have described that 64% of patients with three or more diseases (out of a list of 64) were defined with a triade within the six most prevalent conditions (hypertension, lipid disorder, diabetes, low back pain, osteoarthritis, ischemic heart disease). They suggested that an adjustment and alignment of clinical guidelines for these six conditions would constitute a big step towards an adaptation of guidelines for multimorbid patients. However, these six problems include conditions which might have a very differing impact at patient level (for example low back pain and osteoarthritis) which may hamper the use of standardized guidelines. Moreover, our study has indicated a considerable prevalence of mental health problems and social problems which will also be less easily included in standardized guidelines and programs because they often require tailored and individualized instead of programmed and standardized care.

### Strengths and limitations

The main strength of this study is that the morbidity estimates are derived from chart review rather than automated diagnostic codes [22]. This enabled a comprehensive assessment of multimorbidity including merely problems that were significant for the clinical management of the patient and enabling the inclusion of all problems whereas most studies are obliged to a pre-selection of chronic diseases [21–23]. This practice-based individualized assessment by family doctors provides a comprehensive insight in the day to day presentation of multimorbidity in primary care. The individualized assessment by clinicians may hamper validity of data but first, chart review within primary care has been validated for the assessment of multimorbidity [24] and second, the assessment approach has been harmonized. The results of this study require cautious interpretation mostly because prevalence figures on multimorbidity are highly determined by study design and datasources [20, 21]. Fortin et al. have indicated that prevalence estimates are higher in the primary care setting than in the general population [21, 22]. Moreover, because we were able to include every condition instead of a limited list higher prevalence figures are to be expected [21]. Because the aim of this specific study included an assessment of multimorbidity the way it presents to family doctors we believe our results indicate that the usefulness of guidelines on guidelines for disease combinations will be hampered at the level of clinical practice. A main disadvantage of our method is that detailed clinical review is not feasible for larger samples. The analysis of only 543 patients in two family practices in Belgium should be considered a limitation as is the fact that we only assessed combinations whereas many patients have more than two problems. The generalizability of our results may also be hampered due to the particularly deprived population within the CHC’s and the inclusion of older patients. However, the high morbidity load and complexity of this population should have been suitable to retrieve the combinations which in the end are relevant at practice level in primary care.

## Conclusion

Our results show that patients with multimorbidity often have complex and unique combinations of problems. Low prevalence of disease combinations at practice level and the fact that many patients have more than two problems which influence clinical management make it unlikely that performing trials or developing guidelines for people with specific combinations will ever be useful at the level of clinical practice. The need for an individual approach is further emphasized by the high prevalence of social and psychiatric problems. We need to explore new generic ways and paradigms to approach patients with multimorbidity which allow to tailor care to each individual patient [19].

## Abbreviations

FD: Family doctor
(E)PR: (Electronic) Patient record
SOAP-format: Subjective (S), objective (O) assessment (A) and diagnostic and treatment plans (P)
CIRS: Cumulative illness rating scale
EENT: Eye, ear, nose and throat system
COPD: Chronic obstructive pulmonary disease
NSAIDs: Non steroidal anti-inflammatory drugs
TIA: Transient ischemic attack
CVA: Cerebrovascular accident.
